# Affective Attachments: Women's Suffrage in Austria and the Social Democratic Struggle for Women's Votes in *Die Unzufriedene*

**DOI:** 10.3389/fsoc.2019.00028

**Published:** 2019-05-15

**Authors:** Brigitte Bargetz

**Affiliations:** Department of Political Science, University of Vienna, Vienna, Austria

**Keywords:** women's suffrage, social democracy, affective politics, belonging, agency, discontent, Austria

## Abstract

“Human progress lies in discontent!” was the motto of the Austrian magazine *Die Unzufriedene* (*The Discontented*). It was first published in 1923 as an “independent weekly magazine” designed to reach “all women.” Yet, it was first and foremost a Social Democratic journal, established to socialize women politically and to obtain women's votes outside the Social Democratic purview in the 1923 National Council elections. Since women's suffrage had been established only a few years earlier, the struggle for women's votes was of utmost importance. This essay argues for understanding the journal as a mode and an instrument for the mobilization of affects for Social Democratic ends. Proposing the concept of affective attachments, it shows how the Austrian Social Democratic Workers' Party (SDAP) used the journal in an ambivalent way to affectively address women and to create political moods that would attract them to the party's political agenda.

## Introduction

In her recent book *Crowds and Party*, Dean (2016) calls for reconfiguring the party form as a way to strengthen the emancipatory potential of left-wing politics. The party she envisions explicitly bears the crucial yet often neglected dimension of affect. Within politics, and particularly political parties today, emphasizing affect and emotions is nothing new. However, whether affect and passions are indeed instructive for emancipatory politics, as Dean suggests, remains contested—particularly in light of the rise in right-wing populist affective politics. In the early 2000s, Mouffe already attributed part of the success of right-wing parties to their ability to provide objects that voters could identify with. For Mouffe (2005, p. 25) this does not mean to suggest dismissing passions in politics but, rather, paying attention to and acknowledging passions as both driving forces and as a crucial mode of collective identification, especially during elections:

In voting is an important affective dimension. […] [W]hat is at stake there is a question of identification. In order to act politically, people need to be able to identify with a collective identity which provides an idea of themselves they can valorize. Political discourse has to offer not only policies but also identities which can help people make sense of what they are experiencing as well as giving them hope for the future.

Consequently, emancipatory politics needs to mobilize passions for democratic goals (Mouffe, 2001).

**Figure 1:**
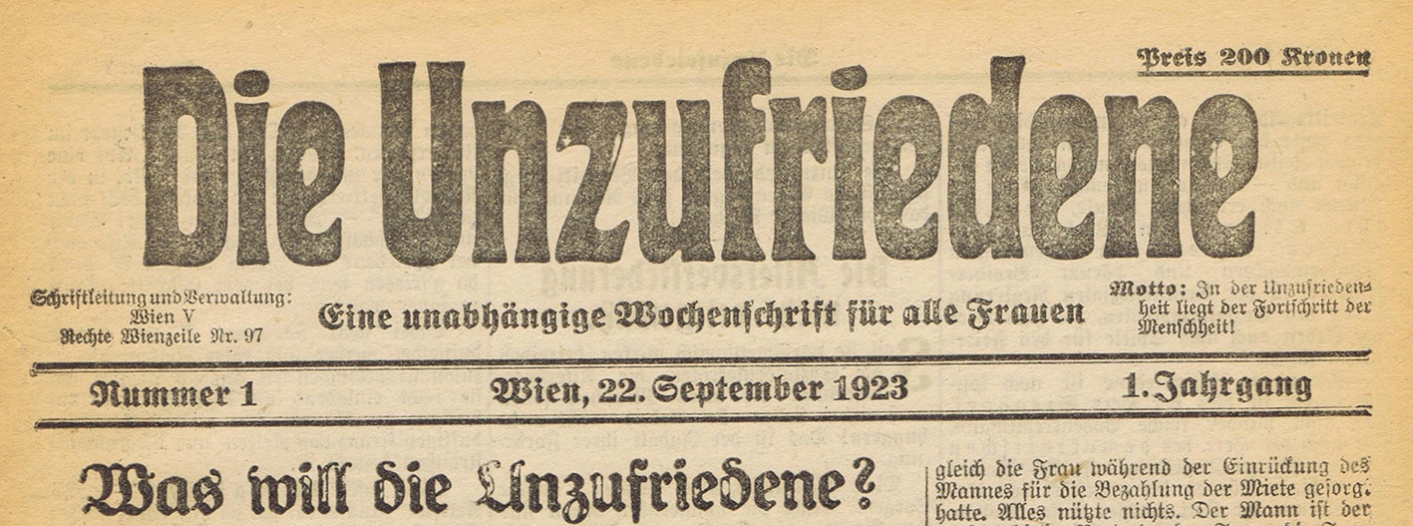
First edition of *The Discontented* (Die Unzufriedene, 1923c) (Source: Association for the History of the Workers' Movement, Vienna; used with permission).

Awareness of the importance of political affects both structures and shapes the early twentieth century Social Democratic magazine *Die Unzufriedene* (*The Discontented*^1^). The magazine, however, does not merely stress the feeling of discontent, as its name suggests. By claiming that “Human progress lies in discontent!”^2^ it also deems this feeling to work in favor of political transformation. The magazine was created in 1923 as an “independent weekly magazine” that sought to reach “all women” (see Figure 1). That being said, it was most of all a Social Democratic “militant magazine” (Hons-Nowotny, 1933, p. 6) or as Bader-Zaar (1996, p. 73) writes, an “easily digestible Social Democratic propaganda newspaper with articles on social legislation […] and selected feminist objectives […] interspersed with stories on housekeeping, fashion, and novels.” It was, after all, established in order to socialize women politically and to obtain women's votes outside the Social Democratic purview in the upcoming National Council^3^ elections. Since women's suffrage had been established only a few years earlier, in 1918, the struggle for women's votes was of utmost importance.

I suggest an affective reading of the magazine, which can help better grasp the complex ways in which affect has been mobilized for Social Democratic ends^4^. For some years now, the “affective turn” (cf. Clough and Halley, 2007; Koivunen, 2010; Bargetz and Sauer, 2015) has attracted and affected a variety of disciplines, amongst others the historical sciences, where the “history of emotions” was introduced “with a great deal of fanfare and frills,” as Frevert (2009, p. 183) put it a few years ago. In my reading I specifically draw upon queer feminist insights on affective politics (cf. Sauer, 1999; Berlant, 2008; Ahmed, 2010; Bargetz, 2014b) and propose the concept of affective attachments. I argue that the magazine is not merely an object of affective identification as Mouffe claims. Rather, it is both an object and a mode of creating a mood for people to vote in favor of Social Democracy. As Flatley (2008, p. 23) asserts, any “kind of political project must have the “making and using” of mood as part and parcel of the project.” The magazine represents an object that helps create, nurture, or gesture toward structures of belonging. It provides a space of recognition and includes moments of caring and sharing, of hope and solidarity. Affective attachments are created and worked upon through the invocation of a social imaginary and the promise of a different future.

Yet, speaking of affective attachments also means investigating how affect is mobilized as a rhetoric of difference for the justification and reproduction of structural hierarchies as well as modes of exclusion and erasure. Historically, affect and emotions have frequently been devalued and delegitimized politically through their association with gender, sex, race, and class. In this manner, affect and emotions prove to be sticky because affect is, as Ahmed (2010, p. 29) writes, “what sticks, or what sustains or preserves the connection between ideas, values, and objects.” Affects are sticky in the sense that multiple differences and hierarchies are attached to them. Affect and emotions are thus a contested battleground. In this manner, the concept of affective attachments asks how (manifold) sentiments are unequally distributed through *The Discontented* in order to create affective investments with and feelings of belonging to Social Democracy.

The concept of affective attachments offers a way for further theorizing an understanding of affective politics and bringing to light how affective registers are deployed for the purpose of political mobilization. Affective attachments emphasize both the connective and sticky mode of affect as well as the affective traces of social hierarchies and differences and how these are re-articulated politically. Developing the concept of affective attachments with regard to *The Discontented*, I also hope to offer more than a way of thinking through political affect. I engage with a magazine that has only rarely been explored in more detail^5^ compared to other Social Democratic magazines from that same era such as the *Kuckuck* (*The Cuckoo*)^6^. Keeping in mind the magazine's aim of mobilizing women, I examine especially the issues that appeared around the elections of the First Republic. My analysis draws on feature and lead articles, the column entitled *Was sich Frauen von der Seele reden* (*Women Speak from the Heart*^7^), as well as letters to the editors and the magazine's responses. All these sections articulate relational dimensions. Conceiving of affect as a mode of relationality and connectivity—attachment, Berlant (2011, p. 13) observes, is a “*structure* of relationality”—these sections are therefore of particular interest in terms of an understanding of affective attachments.

## Situating *the Discontented*

In 1923, the year *The Discontented* was founded, the First Austrian Republic had been existing for only 5 years. The Republic was established after the end of World War I and marked the downfall of the centuries-long Habsburg Empire. In its place, a republican political system was introduced though, with it, came political conflicts and economic problems. The initial coalition between the Social Democratic Workers' Party (SDAP) and the Christian Social Party (CSP) was short-lived. When the coalition collapsed in 1920, the SDAP went into the opposition until 1934, when it was dissolved in the wake of the May Constitution and the emergence of the Austrofascist state^8^. The only exception was Austria's capital. Here, the SDAP won the absolute majority in the 1919 municipal election, paving the way for a strong Social Democratic opposition—what came to be known as Rotes Wien (Red Vienna)—to the conservative national government until 1934.

The end of the Habsburg Monarchy and the founding of the First Republic turned out to be a felicitous moment for women's suffrage—in contrast to the situation 11 years earlier, when Social Democratic women agreed to withdraw their own demand for suffrage in favor of obtaining a universal male suffrage. Still, compared to other European countries, demands for women's suffrage were voiced relatively late in Austria. These “belated demands” (Bader-Zaar, 1996, p. 61) were closely linked to the political circumstances of the time. Women first demanded (active and passive^9^) universal suffrage^10^ during the 1848 revolution and formed associations such as the Vienna Democratic Women's Association (Hauch, 1995a, p. 276; Hauch, 1995b, p. 30f). The situation changed drastically once the revolution was repressed (Hauch, 1995b, p. 38; Bader-Zaar, 1996, p. 61). The political opportunity structures did not favor women or their political struggles and became even less favorable when the 1849 and 1867 legislations prohibited women alongside minors and foreigners to form or join any kind of political association (Hauch, 1995b, p. 39). Demands for women's suffrage only resurfaced in the 1890s following the emergence of the women's movement in the 1880s^11^. In 1893, after three unsuccessful petitions, the Allgemeiner Österreichischer Frauenverein (AÖFV, General Austrian Women's Association)^12^ was permitted—yet only after it was declared as explicitly apolitical association (Hauch, 1995b, p. 44).

## Fear of Women's Votes

Introducing the right to vote for (most) women in Austria in 1918^13^ was not only met with enthusiasm but also with fear and uncertainty, amongst both the Christian Social and Social Democratic camp. The Christian Social Party (CSP) feared that politically conservative and presumably disinterested women would refrain from voting and that the votes of “radical women” would prevail (Zaar, 1998, p. 68). The Social Democratic Workers' Party (SDAP) had been the first to advocate for women's suffrage in the 1890s, integrating this demand into their political program in 1892. In the early twentieth century, however, this goal was subordinated to demands for male universal suffrage. In 1903, the founder of the SDAP, Victor Adler, stated that demanding universal suffrage for men and women at the same time would cause a distraction and would even be “politically foolish” (2. Reichsfrauenkonferenz 1903, qtd. in Ellmeier, 2006, p. 7). When male universal suffrage was finally introduced in 1907, Adler conceded that it had mainly been achieved through “the sacrifice, discipline, understanding, and dedication of our female comrades” who had “voluntarily and self-evidently accepted the tactical necessities of this struggle” (Adler, qtd. in Dohnal, 1988, p. 9)^14^.

Ultimately, it was Social Democrats' anxieties, rather than those of the Christian Socials, that became reality. At the first National Council elections in 1920, women voted as conservatively as they had the year before in the Constituent National Assembly^15^ elections (Zaar, 1998, p. 68). This tendency continued until 1930^16^. Regarding the 1927 National Council elections, Danneberg (1927, p. 14), a Social Democrat Member of Parliament and key politician of the Red Vienna era, stated that “60% of women's votes were bourgeois and 40% Marxist, while the men voted 55:45, respectively.” He concluded that, “Women's suffrage has cost us five mandates” (Danneberg, 1927, p. 14). Social Democratic women, however, continuously rejected such accusations and blame, emphasizing instead the party's overall loss of votes and the need for common struggles (Hauch, 1995a, p. 280).

## Politicizing Dissatisfaction and Disaffection

Against this background, the magazine *The Discontented* was founded to generate an interest for and an attachment to the Social Democrats^17^. With the creation of the *Arbeiterinnen-Zeitung* (ANZ*, Women Workers' Newspaper*, renamed *Die Frau/The Woman* in 1924) in 1892, Social Democrats were already publishing an independent women's newspaper. The *Women Workers' Newspaper*, however, mainly reached women already involved in the party or in trade unions. In order to attract a broader audience, Max Winter launched *The Discontented*. Winter was not only the magazine's founder and first editor-in-chief, he also was the author of numerous social reportages for the Social Democratic *Arbeiter-Zeitung* (AZ, *Workers' Newspaper*) as well as a politician. Another major figure of the magazine was Eugenie Brandl, the magazine's administrative editor. After Winter left the magazine in 1933 at the age of 64, his former colleague Paula Hons-Novotny, who had started as a secretary to the editor in 1927, took his place as editor-in-chief^18^. While in-depth research on the magazine's editors and authors is lacking, it is interesting to note that both male and female authors contributed to *The Discontented*^19^. Following the February 1934 uprisings, the magazine's publication was temporarily suspended. After being politically completely revamped, it reappeared as *Das kleine Frauenblatt: eine unabhängige Wochenschrift für alle Frauen* (*The Little Women's Magazine: An Independent Weekly for all Women*), a political organ of the Austrofascist state.

The idea behind *The Discontented* was to keep the magazine simple and to strike a good balance between politics, education, entertainment, and self-help. It was relatively cheap in comparison to other magazines and newspapers, so as not to burden workers' tight budget. It was published once a week and was successful in reaching a large audience quickly. The first edition consisted of 59,000 copies; 106,400 by the end of December 1923; and 161,400 copies at its peak in 1930 (Furchheim, 2009, p. 2). A survey of 1,320 female factory workers in Vienna in the 1930s (Leichter, 1932) has shown that 21,4% read *Die Unzufriedene* (*The Discontented*), 48,2% read the daily *Das Kleine Blatt* (*The Little Newspaper*), and 28,8% read the daily *Arbeiter-Zeitung* (*Workers' Newspaper*) (Gruber, 1991, p. 89). The high rate of success from the very first issue onwards helped keep the magazine going, even though its initial purpose had only been to secure votes for the 1923 elections.

From a feminist perspective, the magazine's motto—discontent—is highly interesting. Discontent and complaint have been associated with women for many centuries, perhaps even millennia. Almost 2,400 years ago in *Politeia*, his book on the state, Plato demanded “lamenting” be stopped and left for “women” (Platon, 1991, p. 185). *The Discontented* was clearly aware of this trope, as the first issue's description of the magazine's aims suggest. The magazine was not to indulge in “lamenting,” “complaining about fate,” or “ranting” but to “reveal the actual state of affairs” (Die Unzufriedene, 1923c, p. 1). Following Ahmed's (2010) work, *The Discontented* could even be called a “feminist killjoy.” With the figure of the feminist killjoy, Ahmed (2010, p. 65) underlines that critiques of (hetero-)sexism are often devalued by considering those uttering such critique as “killing joy” and therefore standing in the way of others' happiness. Ahmed, however, advocates for the appropriation of the image of the feminist killjoy, which means keeping on exposing (hetero-)sexism and remaining politically uncomfortable. Embodying a feminist killjoy *avant la lettre, The Discontented* not only affirmed but also emphasized discontent as a mode of critique of ongoing gendered oppression. The first edition displays this when it contentiously asks: “Are we not still the oppressed? The slaves of men, factories, at work on the job or at home? Are we not slaves to our backward culture, our legislation?” (Die Unzufriedene, 1923c, p. 1).

By referencing discontent in its name, the magazine adopts a quite militant tone. Grievances must be named, made public, and fought against together. *The Discontented* aims to serve as the “voice” of oppressed women (Die Unzufriedene, 1923c, p. 1). The first issue explains that patriarchy oppresses women and that this oppression is visible in gender relations, economic and cultural conditions, and the state. Women's oppression, as the magazine states, is “reactionary” and prohibits “progress” (Die Unzufriedene, 1923c, p. 1). In this manner and in line with the Social Democratic understanding of progress of that time, the magazine was meant to be a step into the future of a more equal world. *The Discontented* “leads the fight against all that is unjust, ignorant and backward. Human progress lies in discontent” (Die Unzufriedene, 1923c, p. 1). Overcoming patriarchal oppression was therefore both a sign and an expression of democracy.

At times however, this militant tone appears to soften. This is the case when the magazine considers itself less concerned with “letting our fury simply run wild” and more with “showing how to do things better” (Die Unzufriedene, 1923c, p. 1). Moreover, although *The Discontented* emphasizes women's agency, it often frames it less as an opportunity and more as a duty. The lead article in the first issue argues women are obligated to take up this responsibility and closes with the following injunction: “If women are to move forward, you too must be discontent” (Die Unzufriedene, 1923c, p. 1). The mobilization of duty and responsibility, then, seems to refer as much to (ascribed) female attributes as to the Social Democratic idea of developing class consciousness. Subsequent issues continue addressing women in moral terms. In view of the upcoming National Council elections, the magazine raises the so-called “question of conscience”: Evoking feelings of care and responsibility, the magazine addresses its readers and asks “How will you vote?” so that the elections are not followed by “years of remorse” (Die Unzufriedene, 1923d, p. 1)?

**Figure 2:**
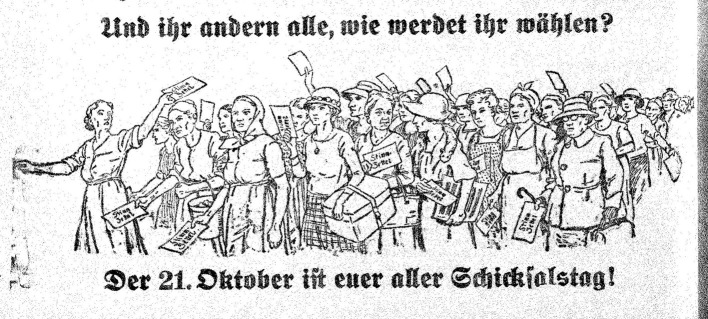
*The Discontented* in the run-up to the 1923 National Council elections: “The 21st of October be your day of fate!” (Die Unzufriedene, 1923d, p. 6) (Source: ANNO. Historical Austrian newspapers and magazines, Austrian National Library; used with permission).

Apparently, this question of conscience may have been especially designed to gain the approval of Christian Social women. This reading is supported by the fact that in an article on 20 October 1923 the “Christian women” (Die Unzufriedene, 1923d, p. 1) is the first one of a total of 32 groups of women to be addressed. Additionally, for instance, Jewish women, German women, Czech women, mothers, servants, factory workers, settlers, blind women, grandmothers, and finally, “all of you” (Die Unzufriedene, 1923d, p. 6; see Figure 2) are being reminded of the reasons for voting Social Democracy. A sense of duty and gratitude, albeit in a more combative tone, is also emphasized by Marie T., a reader from the Austrian town of Klagenfurt:

The Women should think about the fact that the ballot they hold in their hands is a weapon they can use to fight every injustice done unto them. […] Women suffer under anti-working-class politics, and they have the power to change these politics to meet their needs. They must recognize the power of women's suffrage and give their vote to those who have given them this right, who understand their suffering, and who can rectify it (T., Die Unzufriedene, 1923e, p. 5).

**Figure 3:**
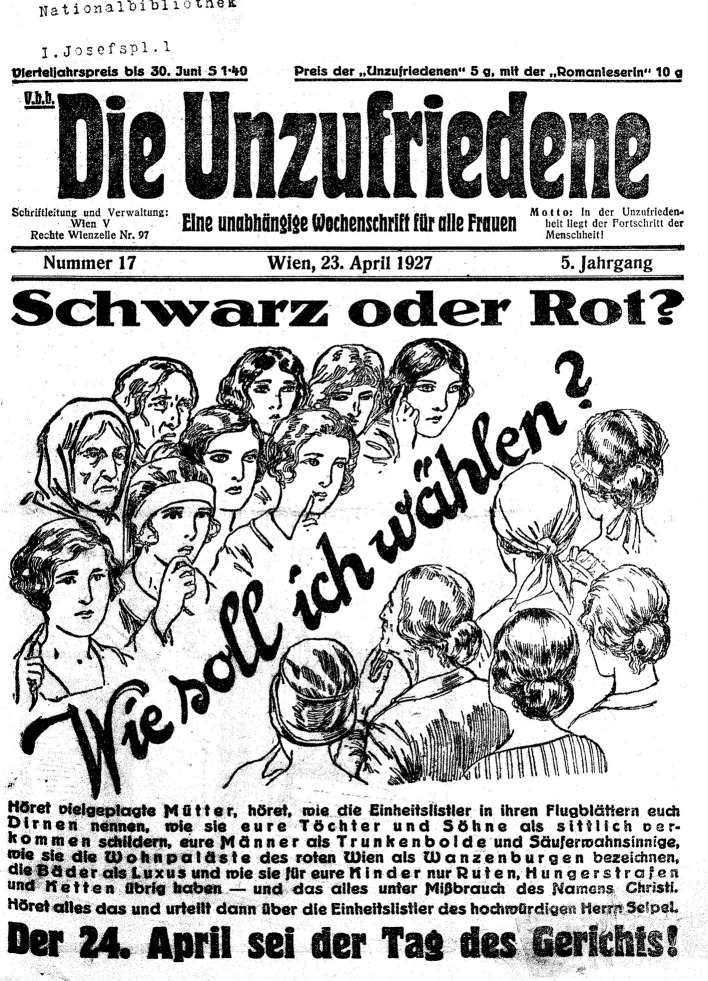
In the run-up to the National Council elections in 1927: “Black or Red. How should I vote? The 24th of April is the Day of Judgment.” Die Unzufriedene (1927b, p. 1) (Source: ANNO. Historical Austrian newspapers and magazines, Austrian National Library; used with permission).

Four years later, in the run-up to the National Council elections of 1927, the magazine again addresses women's responsibility when it tries to persuade its readers to vote for Social Democracy (see Figure 3). One quarter of a double page portrays a bourgeois nuclear family with a servant, meant to represent the past; the rest of the space depicts 8 years of Social Democratic achievements—including the idea of “community,” “common property” and a “laundry room” for women (Die Unzufriedene, 1927a, p. 4f)—as well as a glimpse into a Social Democratic future. The image caption is revealing: “Do you want to choose the past, wife and mother, or the future?” (Die Unzufriedene, 1927a, p. 4f).

*The Discontented* invokes women's affective attachment to Social Democracy through the deployment of anger and dissatisfaction as legitimate political moods. This, however, is only one way of trying to gain their support. Another one is to mobilize gendered sentiments such as gratitude and responsibility—accountability for the fate of Social Democracy, the fate of a democracy that is yet to come.

## The Personal is Political?

The column *Women Speak from the Heart*, where “every woman has a chance to speak” (Die Unzufriedene, 1923b, p. 5), was meant to enable women to raise their (political) voice outside electoral periods. Since its inception, as Winter (1933, p. 2) notes in his essay “Ten Years of The Discontented,” the magazine placed “great emphasis on letting women speak for themselves as much as possible.” He describes the idea behind the column as a way of empowering “women who had never taken up a pen before” or have never had the “courage to do so” (Winter, 1933, p. 2). Indeed, the column became an affective political space for women to express a wide range of fears, worries, and everyday problems; or in Winter's words (1933, p. 2), an “inexhaustible abundance of proposals, suggestions, complaints, and opinions concerning the public state of affairs.” The affective attachment to the magazine and consequently to Social Democracy itself was meant to succeed by demonstrating that the SDAP took women and their everyday activities seriously. One might describe the column with the famous slogan from the late 1960s/early 1970s as “The personal is political,” echoing the longstanding feminist critique of the public/private dichotomy (Patement, 1989). The column allowed the politicization of feminized domesticity and minoritized embodiment. It suggests affective identification both as a part and as basis for political solidarity and thus articulates the potential of deindividualizing and collectivizing experiences. In this vein, the column underscores the political dimensions of the everyday. The everyday, then, is not the insignificant, unrecognized or banal: rather, it is, as Lefebvre (2002 [1971]) emphasizes, an ambivalent assemblage of practices deeply embedded in power relations (Bargetz, 2014a); and the political is “hidden in the everyday” (Kaplan and Ross, 1987, p. 3).

Ultimately, however, *The Discontented* proved ambivalent. Women's everyday life was at best only partially considered political compared to the more “common” sphere of (state) politics. Furthermore, women were believed to lack political consciousness.

In order to elaborate on these two aspects, it is helpful to have a look at the Social Democratic newspaper *Das Kleine Blatt (The Little Newspaper)*, founded in 1927 as a small-scale, inexpensive and sensationalist daily. Modeled after the English penny press, *The Little Newspaper* was designed to offer an alternative to the *Workers' Newspaper* and to rival bourgeois tabloids (Seiter, 1995, p. 52). In an article entitled “Politik, die die Frauen verstehen” (“The Politics Women Understand”), the newspaper's co-editor Pollak (1927, p. 3) emphasizes the combative power of women regarding the 1927 burning of the Palace of Justice^20^. For it was women, she argues, who “above all, originally, vehemently, and from a deep inner feeling of solidarity jumped up and spoke out” (Pollak, 1927, p. 3). However, despite acknowledging women's involvement in these political struggles, the article's title—“the politics women understand”— indicates that women are not only supposed to have a “different” understanding of politics but, also, to only be able to understand particular forms of politics. According to Pollak (1927, p. 3), the forms of politics that women *do* understand are not focused on “discussing the budget,” “central committees,” or “party negotiations.” She claims these are not venues for women's political passions, they do “not captivate women” (Pollak, 1927, p. 3). Rather, women are concerned with “the simple facts of everyday life” and this is “what they have come to learn” (Pollak, 1927, p. 3).

Pollak (1927, p. 3) nonetheless acknowledges the politics women pursue:

And it has been proven that women's feelings—often belittled and waved aside as “sentimental”—can become a powerful moral force. The echo of those Bloody Days in Vienna [the Palace of Justice fire] has never sounded louder than in the hearts of mothers. […] Where the pettiness of everyday life once faded into gossip, into rippling murmurs of women's voices in Vienna: this has now grown into a torrent of voices for a political cause.

Here, Pollak highlights women's agency, challenging long-standing portrayals of women as apolitical, emotional, and irrational (cf. Frye, 1983; Lorde, 1984; Prokhovnik, 1999; Sauer, 1999). Such assumptions still echoed when women demanded the right to vote. Women were supposed to be closer to nature and deemed emotionally “too impulsive, too nervous” (Initiative 70 Jahre Frauenwahlrecht, 1989, p. 9) to be able to handle voting. Because of this, they were to be relegated—physically and mentally—to the private sphere. Their “brains” were said to be “lighter and more finely organized” (Ettel, 1890; cf. Bader-Zaar, 1996, p. 66f; Hauch, 1995b, p. 46f) and they were considered to represent a “natural lack of defensive strength, depth and prudence in judgment, determination in will and perseverance in action” (Schindler qtd. in Bader-Zaar, 2006, p. 1007). For some, women's political engagement was thought to negatively influence the family as much as the state (Ettel, 1890, p. 19).

Pollak counters this by emphasizing the political force of affect, which alludes to a sense of being affected and affecting others (Spinoza, 1999). In her view, the affective investments of the women protesting have fostered the 1927 uprisings. Unlike other commentators, Pollak does not discredit feelings. She contrasts them to what she labels “idle gossip (Pollak, 1927, p. 3) and what she relegates to (these) women's past. In this manner, women's involvement and actions make explicit affects' and feelings' force of solidarity which, for Pollak, has emerged during protests. Women are affectively addressed by being recognized as feeling political subjects.

However, Pollak also stresses that women's political engagement differs from commonplace politics, which matters in terms of their mobilization. Women's politics “must be human, comprehensible, and life-oriented,” that is a politics “that captivates and engages them” (Pollak, 1927, p. 3). While Pollak virtually de-privatizes and acknowledges affect and feelings as political forces, a familiar mode of gendered denial simultaneously comes to light: because women are supposed to pursue “different” political interests and primarily function as a moral force, their ability to engage in “politics as a vocation,” to speak with Weber (1946 [1919]), is ultimately made impossible. Accordingly, women appear to be imprisoned in everyday life, as Lefebvre (2002 [1971]) once put it. The personal is indeed political but only in very specific ways.

The specific female politics that Pollak points to also appear in *The Discontented's* response to a letter from I.C. in October 1924. The latter had turned to the magazine, searching for advice regarding difficulties organizing in an “industrial city in the province” (Die Unzufriedene, 1924b, p. 4). While the writer lists several issues that impede organization—ranging from lack of time, disinterest, objections from husbands or wives, to sensitivities between women—the magazine's response merely focuses on the very last. The “organizational misery in the provinces” (Die Unzufriedene, 1924b, p. 4) becomes individualized by the magazine's response. On the one hand, it is reduced to a feeling of overwhelming “self-love,” from which the “great mass of workers” needs to be freed through education; only such education, the magazine claims, would teach the value of “solidarity” and a “sense of belonging” (Die Unzufriedene, 1924b, p. 4). On the other hand, the (presumably female) writer of the letter is addressed in her emotional capacities. The magazine encourages I.C. to act in a “conciliatory, mediating and enlightening” manner and to unite “the quarrelsome sisters, who attack one another with jealousy” (Die Unzufriedene, 1924b, p. 4).

## Enlightenment and Patronizing

By emphasizing dissatisfaction and political agency, the magazine encourages women to take an active part in politics (Die Unzufriedene, 1923a). Yet, women are also considered ignorant, lacking critical political awareness and, most of all, in need of help. *The Discontented* was meant to educate women so that they could develop political consciousness. In the very same gesture where women are called up to participate in politics, they are denied political agency. Predictably, following this logic, women are assigned a subordinate position within the ongoing political power structures, invoking the well-known liberal dichotomy of “male rationality” and “female irrationality.”

In the aforementioned essay “Ten Years of The Discontented,” Winter (1933, p. 2) looks back at the historical moment when the magazine was first created: “At that time it was necessary to approach the many tens of thousands perhaps even hundreds of thousands of unconscious working women.” For that reason, the magazine was dedicated to “help them with the difficulties of voting” (Winter, 1933, p. 2). In a similar manner, on 20 October 1923, the day before the election, *The Discontented* responds to the question of how to vote as follows: “We will try to help everyone so that they can find their way” (Die Unzufriedene, 1923d, p. 1). This mixture of addressing women and promising them help and enlightenment as a mode of affective mobilizing is just as striking 7 years later. Welcoming its new readers in 1930, *The Discontented* describes its purpose as wanting to be “an aid” to “bring a little political enlightenment” and to “help a bit to think about why our circumstances are so unpleasant” (Die Unzufriedene, 1930a, p. 1). Still, this pedagogical emphasis was not only a gendered issue but symptomatic for the contemporary Social Democratic efforts and hopes to educate workers in order to fuel political transformation. As Otto Glöckel, a leading Social Democratic pedagogue and politician, puts it: “Once people have the courage to gain knowledge, they must become socialists” (Workers-Newspaper, 5 December 1930: 8, qtd. in Gruber, 1991, p. 87).

Providing aid and education for the supposedly ignorant woman was also a topic at the 1920 party congress of the SDAP: “For the enlightenment among those women who cannot be reached through our organization and the party press, a popular weekly paper is necessary […] one that illuminates all problems of work, families and society in an easy-to-understand manner” (qtd. in Billeth, 2003, p. 8). This assessment was widely shared. August Bebel—co-founder of the Sozialdemokratische Arbeiterpartei Deutschlands (SDAP, German Social Democratic Workers' Party) who had pushed for the universal suffrage for women and men together with Clara Zetkin, a central figure of the proletarian women's movement in Germany—emphasizes the need to educate ignorant women in his 1878 book *Die Frau und der Sozialismus* (*Woman and Socialism;* Bebel, 2014 [1910], p. 608f):

Even progressive people argue that it would be dangerous to enfranchise women because they are conservative by nature and are susceptible to religious prejudices. But these arguments are true to some extent only, so long as women are maintained in ignorance. Our object must therefore be to educate them and to teach them where their true interest lies.

In the pages of *The Discontented*, the assumption of women's lack of knowledge reads as follows: Regarding a protest of civil servants on 10 September 1925, a contributor to the newspaper, M.S., urges these “dear women” not to remain “ignorant”: “You working women, do not remain in dullness and dumbness” (Die Unzufriedene, 1925, p. 1f). Similar beliefs shape the responses published in the column *Women Speak from the Heart*. By judging readers' insights voiced in their letters as right or wrong, the magazine takes up the role of a benevolent schoolmaster. For instance, on 22 November 1924 the magazine states, most generously: “Your line of reasoning is totally correct, if all housemaids would think that way and move in the right direction, namely to join the organization, nothing more would stand in the way of it becoming reality” (Die Unzufriedene, 1924a, p. 4).

In this same vein, the following article published for the tenth anniversary of *The Discontented* is noteworthy:

Max Winter has introduced thousands and thousands of proletarian women to socialism and to the Social Democratic Party through the convincing and noble power of his speeches and writing. And if the Social Democracy of Austria of today does not regret standing up for women's suffrage, it is first and foremost thanks to Max Winter and *The Discontented*, which has become, as Winter's deceased friend Friedrich Austerlitz^21^ once described him, as “the true awakener of working-class girls and workers' wives, whom he educates to read and think, and above all helps to educate themselves” (W. R., 1933, p. 3).

Notwithstanding the comprehensibly euphoric tone in view of a jubilee number, the references to the “noble power” and the “true awakener” that are used to describe the figure of the “founding father” Winter as well as the invocation of deep gratitude that his deeds are meant to require, still seems oddly patronizing.

Adelheid Popp, editor of the *Women Workers Newspaper*, at the time already *The Woman*, member of the National Council, and a central figure of the Social Democratic women's movement, was more cautious. Prior to the 1930 elections, she writes that “politically untrained women” had allowed themselves to be “confused” by the Christian Socials in the last election. However, by participating in the 1930 Social Democratic referendum regarding improvements in unemployment, age and invalidity insurance (Die Unzufriedene, 1930b, p. 2) they have shown to have become “more mature” (Popp, 1930, p. 2). And she continues: “You, the women, hold the fate of the working class and thus of Austria—that is your own destiny—in your hands” (Popp, 1930, p. 2).

## Affective Attachments and the Ambivalent Dynamics of Mobilizing

*The Discontented* had set off to mobilize passions for Social Democratic concerns and to create a political mood for voting Social Democracy especially—though not exclusively—for women. Speaking of affective attachments, makes it possible to read the magazine as both a site of political longing and belonging. In her engagement with U.S. publics and politics from the nineteenth century onwards, Berlant (2008, p. x) identifies an affective space as a “space of attachment and identification that is not saturated merely by ideological or cognitive content but is also an important sustainer of people's desires for reciprocity with the world.” In a similar manner, *The Discontented* represented a mediator for political hopes and promises as well as a possibility for affective identification with the Social Democratic Party. It was meant to create political bindings and attachments—both to the party and among the readers. Affective attachments emphasize that political engagement not only involves political interests but also how these interests come to and ultimately do matter as world-building practices. In this sense, the magazine also proved to be a medium of affection (Spinoza, 1999; cf. Deleuze and Guattari, 1987, p. xvi). Taking the everyday seriously and even more so women's everyday feelings as aspects of the political, the magazine aimed at opening up new political spaces of resonance. Discontent and anger epitomized political vehicles and were politicized along with women's struggles against multiple exclusions and exploitative conditions. Because the magazine did not dismiss voicing discontent but recognized it as a political force, it enforced agency and empowerment. As manifestation of discontent and dismay as well as of belonging and solidarity *The Discontented* expressed what I would call “feeling politics” (Bargetz, 2014b, p. 119): that is a sense that politics and power also circulate through feelings and are affectively perceived in the temporalities of the everyday.

That notwithstanding, the political recognition and appreciation of women, as well as the relation to affect and feelings ultimately remained ambivalent within the magazine. In *Why We Oppose Votes For Men*^22^ (1915), American writer, poet, and suffragist Duer Miller (2004 [1915], p. 64) writes:

Because men are too emotional to vote. Their conduct at baseball games and political conventions shows this, while their innate tendency to appeal to force renders them particularly unfit for the task of government.

Of course, this reference to men's overtly and overly emotional behaviors as justification for disqualifying them for dealing with politics is an ironic reaction to women's assumed emotionality and irrationality. Still, the trope of female emotionality and ignorance to explain and justify women's limited political agency was also routinely deployed in *The Discontented*. Although Bader-Zaar emphasizes that “women's alleged ‘inherent' qualities” (Bader-Zaar, 1996, p. 63) have also been placed in a positive and empowering light by Austrian suffragists, even by those in different political camps, these devaluing and minoritizing tropes were widely shared and politically effective. Women were addressed more often morally than politically. Their alleged female mode of existence was read as a form of political ignorance and helplessness. Within *The Discontented*, this was especially reflected in a wide range of patronizing gestures. Women were deemed less political subjects with agency and more often rendered objects.

To conlude, affective attachments are not neutral modes of mobilizing but remain deeply embroiled in (gendered) relations of power and domination. Trying to understand how affective attachments function, therefore, requires attending the complex and complicated mechanisms of affective legacies as well as their multifaceted re-articulations within the historical present.

## Author Contributions

The author confirms being the sole contributor of this work and has approved it for publication.

### Conflict of Interest Statement

The author declares that the research was conducted in the absence of any commercial or financial relationships that could be construed as a potential conflict of interest.

## Footnotes

^1^Throughout the article I translate the periodical's name *Die Unzufriedene* with *The Discontented* to keep with the magazine's telling name.

^2^All translations are by the author.

^3^The National Council is the legislative assembly.

^4^This paper is based on a short essay published in German (Bargetz, 2018).

^5^For some insightful master's theses cf. Billeth, 2003; Lillich, 2004; Gruber, 2007; Furchheim, 2009; Kraus, 2016.

^6^The Social Democratic magazine *Der Kuckuck* (*The Cuckoo*), published between 1929 and 1934, was one of the first “modern picture magazines in Austria” (Riesenfellner and Seiter, 1995, p. 7) and was meant to compete with nonpolitical mainstream boulevard weeklies through explicitly addressing a younger (working-class) audience.

^7^I adopt Gruber's (1991, p. 88) translation of the column's name.

^8^On 12 February 1934, the police had started searching for weapons in the Social Democratic party secretary in Linz. As a reaction, members of the Social Democratic Republican Schutzbund, the paramilitary organization of the SDAP that had been forbidden the year before, opened fire. The uprising soon spread to other industrial towns in Austria, also to Vienna. Already the year before, then Christian Social chancellor Engelbert Dollfuß had used the resignation of all three presidents of the National Council to declare the elimination of the parliament and to install an authoritarian government. In May 1934, the Dollfuss regime decided on a new constitution, which manifested the authoritarian state.

^9^While active suffrage refers to the right to vote, passive suffrage designates the right to be nominated for election.

^10^Officially, suffrage was not limited to men then. It rather referred to an informal rule, as Hauch (1995b, p. 30) stresses, where women's alleged political immaturity seems to have made it unnecessary to even explicitly exclude them.

^11^Still, it is necessary to acknowledge the differences between bourgeois-liberal, Social Democratic and the autonomous suffragette movements (Bader-Zaar, 2006, p. 1007; Hauch, 1995b, p. 44).

^12^Existing from 1893 to 1919, the association's aim was to represent an autonomous position, without links to any of the political parties. Despite this, it did collaborate with the Social Democrats from time to time, but maintained a strict separation from the bourgeois Christian Social camp. (http://www.fraueninbewegung.onb.ac.at/Pages/OrganisationenDetail.aspx?p_iOrganisationID=8675071) (accessed April 2, 2019).

^13^Sex workers obtained this right only five years later in 1923. Some women already had the right to vote a few decades earlier (Bader-Zaar, 1996, p. 61). After 1849 in the Austrian region of the Habsburg monarchy, some women were allowed to vote—actively, but mostly not passively. Suffrage was then based on property and not on sexual difference (Initiative 70 Jahre Frauenwahlrecht, 1989, p. 2). In this sense, the universal suffrage for men in 1905 was even a drawback for some (privileged) women.

^14^This approval, however, was a contested issue. For instance, at the 1903 Social Democratic Women's Conference Therese Schlesinger advocated fighting for women's suffrage while Charlotte Glas-Pohl supported the party line (Bader-Zaar, 2006, p. 1017). Again in 1905, at the Vienna General Party Conference of the SDAP, Adelheid Popp declared that it was not the right time to engage in the struggle for women's suffrage. However, she did emphasize that Social Democratic women were deeply convinced of the backing they would receive from the men as the “first spokesmen and […] pioneers”, once they were “liberated from political servitude” (Bader-Zaar, 2006, p. 1018). In Germany, Clara Zetkin and August Bebel criticized postponing the demand for women's suffrage as too modest and for not taking advantage of the political opportunities (Hauch, 1995b, p. 363).

^15^In February 1919, elections were held for the Constituent National Assembly, which shortly after, in November 1920, was replaced by the Austrian Parliament (consisting of the National Council and the Federal Council).

^16^These insights were possible, since men's and women's votes were placed in differently colored envelopes since the 1920s: light gray for men and blue-gray for women (Bader-Zaar, 1996, p. 71).

^17^According to Bader-Zaar (1996, p. 70), the main question during the First Republic was not the women's right to vote, but rather finding ways “to manipulate women voters to the party's advantage”. On these grounds, some Austrian provinces with a high Christian Social electorate like Styria, Carinthia, Tyrol and Vorarlberg introduced compulsory suffrage.

^18^She is also known as Hons or Novotny, her maiden name.

^19^A complete table of contents of *The Discontented* can be found under the following link that is provided by the Austrian National Library https://www.onb.ac.at/fileadmin/user_upload/1_Sitemap/Forschung/Ariadne/PDF_Inhaltsverzeichnisse/Die_Unzufriedene_inhalt.pdf (accessed April 2, 2019).

^20^On 15 July 1927, during protests in Vienna, the Palace of Justice was set aflame. The protesters demonstrated against the acquittal of three members of a right-wing veterans association (Frontkämpfervereinigung). They had been accused of killing two innocent people in clashes with the paramilitary Republican Schutzbund in January of that year in Schattendorf, a small village in the province of Burgenland.

^21^Friedrich Austerlitz was a member of the National Council and editor-in-chief of the *Workers' Newspaper*.

^22^The text appeared as a response to the 1894 pamphlet *Some Reasons Why We Oppose Votes for Women* written by the National Opposed to Women's Suffrage (NAOWS) (Garber, 2014).
